# Broadening the Voltage Window of 3D-Printed MXene Micro-Supercapacitors with a Hybridized Electrolyte

**DOI:** 10.3390/molecules29061393

**Published:** 2024-03-20

**Authors:** Xin Jiang, Haowen Jia, Xuan Chen, Jiajia Li, Yanling Chen, Jin Jia, Guangzhen Zhao, Lianghao Yu, Guang Zhu, Yuanyuan Zhu

**Affiliations:** 1 Key Laboratory of Spin Electron and Nanomaterials of Anhui Higher Education Institutes, Suzhou University, Suzhou 234000, China; jiangxinty@163.com (X.J.); cxuan0221@163.com (X.C.);; 2 School of Mechanics and Optoelectronic Physics, Anhui University of Science and Technology, Huainan 232001, China

**Keywords:** MXene, micro-supercapacitors, high voltage, aqueous electrolytes, ethylene glycol

## Abstract

The burgeoning demand for miniaturized energy storage devices compatible with the miniaturization trend of electronic technologies necessitates advancements in micro-supercapacitors (MSCs) that promise safety, cost efficiency, and high-speed charging capabilities. However, conventional aqueous MSCs face a significant limitation due to their inherently narrow electrochemical potential window, which restricts their operational voltage and energy density compared to their organic and ionic liquid counterparts. In this study, we introduce an innovative aqueous NaCl/H_2_O/EG hybrid gel electrolyte (comprising common salt (NaCl), H_2_O, ethylene glycol (EG), and SiO_2_) for Ti_3_C_2_T*_x_* MXene MSCs that substantially widens the voltage window to 1.6 V, a notable improvement over traditional aqueous system. By integrating the hybrid electrolyte with 3D-printed MXene electrodes, we realized MSCs with remarkable areal capacitance (1.51 F cm^−2^) and energy density (675 µWh cm^−2^), significantly surpassing existing benchmarks for aqueous MSCs. The strategic formulation of the hybrid electrolyte—a low-concentration NaCl solution with EG—ensures both economic and environmental viability while enabling enhanced electrochemical performance. Furthermore, the MSCs fabricated via 3D printing technology exhibit exceptional flexibility and are suitable for modular device integration, offering a promising avenue for the development of high-performance, sustainable energy storage devices. This advancement not only provides a tangible solution to the challenge of limited voltage windows in aqueous MXene MSCs but also sets a new precedent for the design of next-generation MSCs that align with the needs of an increasingly microdevice-centric world.

## 1. Introduction

The ushering in of the 5G era marks a transformative period in telecommunications, characterized by an exponential increase in data speeds and connectivity that drive the need for a new generation of microelectronic devices. These devices are not only required to be miniaturized, lightweight, and flexible [1,2,3] but also to have reliable power sources that can keep pace with the enhanced functionality and prolonged operation times demanded by 5G applications. Microelectronic power sources, therefore, face the multifaceted challenge of integrating safety, cost-effectiveness, small form factors, mechanical flexibility, and exceptional performance. In this context, MXene-based micro-supercapacitors (MSCs) have emerged as a formidable solution, leveraging the intrinsic properties of MXene materials, such as their remarkable electrical conductivity and atomically thin 2D structure [4,5,6,7]. These properties are pivotal in advancing the charge storage capabilities of MSCs, enabling them to meet the stringent energy demands of 5G-powered devices. MXene MSCs, with their planar configurations, offer a significant advantage in enhancing energy density and device integration, making them ideally suited to power the compact and flexible electronics that are at the heart of the 5G revolution. Such MSCs are envisioned to be integral to a wide array of applications, from wearable technologies to implantable medical devices, where traditional bulky power sources are no longer viable. However, traditional techniques for fabricating MSCs, such as inkjet and screen printing, are hampered by their complexity, cost, and limitations in precision [8,9,10]. These methods are often unable to achieve the required fine-tuned microarchitectures without compromising performance or scalability. In stark contrast, 3D printing technology emerges as a game-changer. It is celebrated for its unparalleled design versatility, rapid prototyping, and high fabrication efficiency [11,12,13,14,15]. Notably, 3D printing affords unprecedented control over the microstructures of MSCs, enabling the precise manipulation of electrode geometries and pore architectures that are critical for optimizing performance. This level of precision and customization is essential for fully harnessing the potential of MXene materials in the context of evolving 5G technologies. The integration of 3D printing technology in the development of MXene MSCs represents a leap forward in the design and manufacture of power sources for next-generation microelectronics. It aligns with the forward-thinking paradigm of the 5G era, offering a scalable path to creating advanced energy storage systems with the sophistication and adaptability to meet the burgeoning demands of an interconnected world.

Despite significant strides in the advancement of micro-supercapacitors (MSCs), one of the most critical challenges that persistently hampers their broader application is the low energy density, an issue that stands at the forefront of contemporary research in the field. The fundamental relationship governing energy storage, E = 1/2CV^2^, elucidates that both the voltage window (V) and capacitance (C) serve as crucial determinants of energy density (E). This relationship has been substantively validated and explored across numerous studies [16,17,18,19,20]. To push the boundaries of MSCs toward levels comparable to those of bulkier counterparts, a considerable body of research has been dedicated to the synthesis of electrode materials with superior performance characteristics. These efforts have resulted in notable improvements in the specific capacitance and cycling stability of supercapacitors. Yet, the role of the electrolyte, a component that is equally essential, has been relatively underemphasized. The electrolyte is not merely a passive medium for ion transport; it plays an active and dynamic role in defining the operational voltage window and enhancing the charge storage mechanisms at the electrode–electrolyte interface [21,22,23]. Recognizing the electrolyte as a key player in this energy interplay, it is imperative to explore innovative electrolyte compositions that can not only withstand higher voltages but also facilitate rapid and efficient ion transport.

Typically, MSCs employ three types of electrolytes: aqueous (e.g., potassium hydroxide, sulfuric acid, and sodium sulfate) [24,25], organic (e.g., carbonates, acetonitrile, ethylene carbonate, ethylene, and propylene carbonate) [25,26], and ionic liquids (e.g., EMI-TFSI, and BMI-PF6) [27,28]. Although organic electrolytes exhibit a wider voltage window (often exceeding 2 V), they are usually toxic and pose numerous safety hazards, particularly to human health. Ionic liquids, with their broad voltage windows (2–4 V), are often prohibitively expensive and toxic and sometimes require blending with organic solvents to achieve optimal performance [29,30,31,32,33].

In contrast, aqueous electrolytes are highly regarded for their safety, low cost, high ionic conductivity, and environmental benignity. These aspects render them particularly attractive for the development of sustainable and eco-friendly microenergy storage devices [22,34,35,36]. Yet, their Achilles’ heel lies in the limited electrochemical stability window—symmetric devices ≤1 V or asymmetric devices ≤2 V with aqueous electrolytes—which severely constrains the energy density achievable in MSCs [37]. To overcome this limitation, “water-in-salt” (WIS) electrolytes have been introduced, extending the voltage window significantly. However, the WIS technology brings about a trade-off, notably in the form of increased electrolyte costs [22,37,38,39]. Furthermore, WIS electrolytes typically exhibit higher viscosity and lower ionic conductivity compared to their diluted counterparts, which hampers electrochemical performance and limits their practical applications [40,41].

The quest for an optimal electrolyte solution continues as researchers strive to balance the trade-offs between voltage window, safety, cost, and performance. Herein, we introduce a hybridized NaCl/H_2_O/EG gel electrolyte comprising low-concentration NaCl salt, ethylene glycol (EG), and SiO_2_, which significantly amplifies the operational voltage of 3D-printed MXene MSCs, thereby elevating their energy density. Compared to the 1 m (mol Kg^−1^) NaCl/H_2_O system, the MXene MSCs fabricated with the 1 m NaCl/H_2_O/EG hybrid aqueous gel electrolyte exhibited an expanded voltage window of up to 1.6 V, a high specific capacitance of 329 mF cm^−2^, and an elevated energy density of 116.8 µWh cm^−2^. The augmentation of active material loading via 3D printing yielded an impressive energy density of approximately 675 µWh cm^−2^, demonstrating substantial potential for practical applications within the microelectronic realm.

## 2. Results and Discussion

### 2.1. Material Fabrication and Characterization

**Figure 1:**
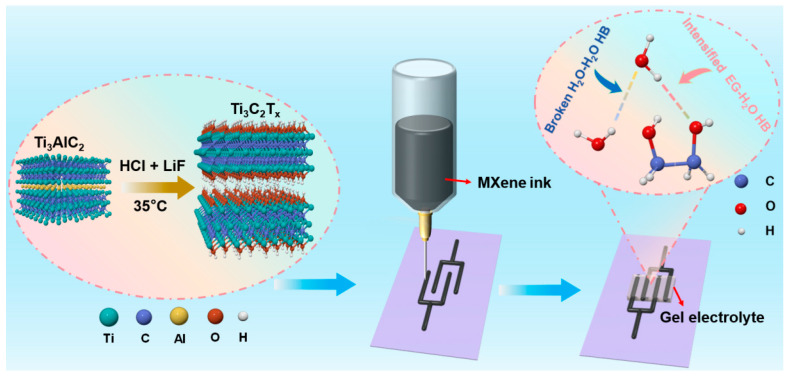
Schematic illustration of the fabrication protocol for MXene MSCs.

**Figure 2:**
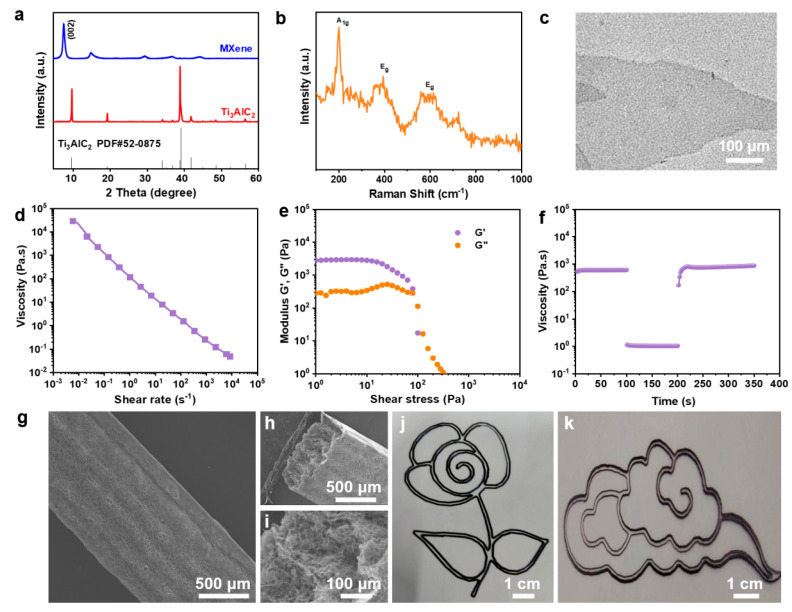
Characterization of MXene ink: (**a**) XRD patterns; (**b**) Raman spectrum; (**c**) TEM image; (**d**) rheological behavior depicting the apparent viscosity as a function of shear rate; (**e**) oscillatory rheology illustrating the relationship between storage modulus (G′) and loss modulus (G″) with oscillatory strain; (**f**) viscosity alterations of the ink under dynamic shear rate conditions; (**g**) lateral scanning electron microscopy image of a six-layer MXene electrode; (**h**,**i**) cross-sectional SEM images of a six-layer electrode at varied magnifications; (**j**,**k**) patterns printed with MXene ink, including floral and auspicious cloud configurations.

The electrochemical performance of MXene MSCs was substantially enhanced through the fabrication of MXene microelectrodes via 3D printing technology, followed by the strategic design of a NaCl/H_2_O/EG hybrid gel electrolyte (Figure 1). Initially, Ti_3_C_2_T*_x_* MXene was synthesized by selectively etching the Al layers from Ti_3_AlC_2_, followed by the acquisition of a printable MXene ink through centrifugation techniques (Figure S1). This ink was then utilized to 3D print customizable interdigitated MXene microelectrodes on a PET substrate. The XRD analysis of MXene, depicted in Figure 2a, revealed pronounced characteristic (002) diffraction peaks of Ti_3_C_2_T*_x_*, indicating the retention of its crystalline structure. The Raman spectrum of Ti_3_C_2_T*_x_*, as shown in Figure 2b, displayed the vibrational fingerprint of the molecule without the presence of TiO_2_ peaks, thus confirming that Ti_3_C_2_T*_x_* was not oxidized. Furthermore, the MXene nanosheets exhibited a quintessential 2D structure with micrometer-scale lateral dimensions and nanoscale thickness [42], as evidenced in Figure 2c.

The rheological properties of inks are pivotal for achieving the precise deposition and shape fidelity required in advanced 3D printing applications. The viscosity behavior of our tailored MXene-based ink under varying shear rates was thoroughly investigated to ascertain its suitability for extrusion-based printing technologies. As depicted in Figure 2d, our results demonstrate a marked decrease in viscosity with an increase in the shear rate, a characteristic indicative of shear-thinning behavior that is typical of non-Newtonian fluids. This rheological attribute is essential for the seamless extrusion of the ink through the printing nozzle while ensuring rapid solidification upon deposition to maintain the intended geometry. A further examination of the viscoelastic nature of the MXene ink was conducted through oscillatory or dynamic rheological analysis at a fixed frequency of 1 Hz. The resulting data, presented in Figure 2e, reveal the ink’s ability to transition from a gel-like state with a dominant storage modulus (G′) to a sol-like state with a predominant loss modulus (G″) as oscillatory stress increases. This transition underscores the ink’s capability to maintain a stable form post-extrusion due to a higher storage modulus (G′) but flow smoothly under the stress of extrusion when the loss modulus (G″) becomes more pronounced. To emulate the actual conditions of 3D printing extrusion, a peak hold rheological test was performed, as illustrated in Figure 2f. In this test, the MXene ink underwent a prolonged low shear rate of 0.1 s^−1^ for 100 s to simulate the ink’s residence time within the nozzle, before a sudden increase to 100 s^−1^ for another 100 s to mimic the extrusion process. A notable decrease in viscosity was observed upon the shear rate increase, which is advantageous for the extrusion process. Crucially, upon returning the shear rate to 0.1 s^−1^, the viscosity rapidly recuperated to its original value, confirming the ink’s robust structural recovery post-extrusion and its suitability for layer-by-layer construction without compromising the final printed structure’s integrity. The exceptional rheological properties and recovery behavior of the MXene ink facilitated the successful 3D printing of intricate MXene microelectrodes, as exemplified by the planar structures shown in Figure S2. The ink demonstrated excellent layer-to-layer adhesion and structural definition, allowing the construction of complex multilayered architectures, with up to six layers printed without any noticeable deformation or loss of definition. Collectively, these findings not only elucidate the intricate rheological behavior of the MXene ink under various shear conditions but also reinforce its potential as a highly versatile material for the additive manufacturing of microscale devices with precise geometrical and functional specifications. Such advanced material formulations are pivotal for the progression of 3D printing technologies and their adaptation to the manufacturing of next-generation electronic components. The analysis of the multilayered planar (Figure 2g) and cross-sectional (Figure 2h) images revealed an average layer thickness of approximately 154 nm. Further magnification of the cross-section exposed a porous architecture, enhancing the ion transport rate while significantly reducing the electrode’s volumetric density (Figure 2i). Based on these favorable characteristics, various 2D planar patterns were adeptly printed with high geometric precision directly from the MXene ink (Figure 2j,k).

### 2.2. Characterization of NaCl/H_2_O/EG Hybrid Electrolytes

**Figure 3:**
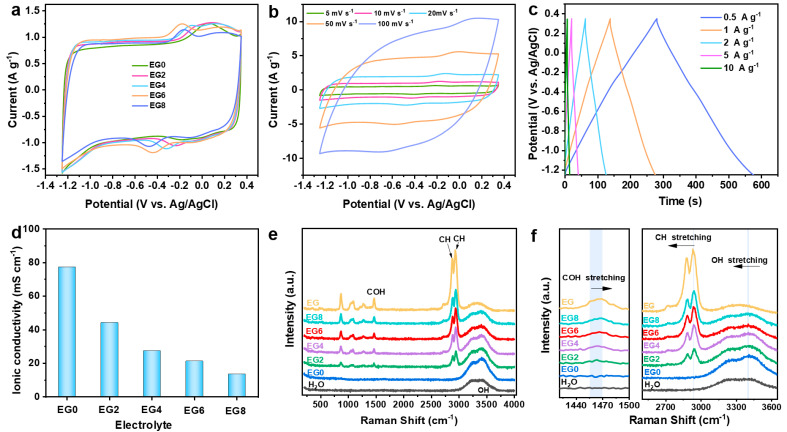
Characterization of NaCl/H_2_O/EG hybrid electrolytes: (**a**) CV curves of MXene electrodes in diverse electrolytes at a scan rate of 10 mV s^−1^; (**b**) CV curves and (**c**) GCD profiles of MXene electrodes using EG6; (**d**) ionic conductivities of the electrolytes at ambient temperature; (**e**) Raman spectra and (**f**) the amplified view within the wavenumber ranges of 1420–1500 cm^−1^ and 2500–3650 cm^−1^ of different electrolytes.

To enhance the electrochemical performance of MXene electrodes, we engineered a series of 1 m NaCl/H_2_O/EG hybrid electrolytes (Figure S3). To elucidate the impact of the NaCl/H_2_O/EG hybrid electrolytes on the electrochemical properties of MXene, we conducted a series of three-electrode electrochemical evaluations, using MXene as the working electrode, platinum foil as the counter electrode, and Ag/AgCl as the reference electrode, with varying compositions of the NaCl/H_2_O/EG hybrid electrolytes. As the content of EG increased, a decrement in the polarization extent was observed in the CV profiles; however, an excess in the EG content resulted in a diminution of the capacitance of the MXene electrodes (Figure 3a). Optimally, the electrochemical performance of the MXene electrodes was found to be superior when utilizing the EG6 electrolyte. Figure 3b,c depict the CV curves at various scan rates and GCD profiles at different current densities, respectively, for the MXene electrodes using the EG6 electrolyte. The electrodes exhibited a broadened electrochemical voltage window of up to 1.6 V (−1.25 V to 0.35 V vs. Ag/AgCl). At a scan rate of 5 mV s^−1^, a specific capacitance of 103.8 F g^−1^ was achieved, which, even at an augmented rate of 100 mV s^−1^, retained a specific capacitance of 62.16 F g^−1^, preserving 60% of its capacitance. Furthermore, the electrodes delivered a high specific capacitance of 99 F g^−1^ at a current density of 0.5 A g^−1^, which, upon elevation to 10 A g^−1^, retained a commendable specific capacitance of 50.20 F g^−1^, accounting for 50% retention, thereby demonstrating exceptional rate capability. Additionally, conductivity assessments of different NaCl/H_2_O/EG hybrid electrolytes indicated a gradual decrease in conductivity with an increase in the EG content; however, the EG6 variant maintained a conductivity of 21.4 mS cm^−1^ (Figure 3d), which surpasses that of several recently reported electrolytes, such as 2 M Zn(OTF)_2_ at 6.5 mS cm^−1^ [43], 1 M Na_2_SO_4_/H_2_O/50% EG at 11 mS cm^−1^ [44], and EM-5Li-Na (5 m LiFSI in [EMIm]FSI with 0.16 m NaTFSI additive) at 2.6 mS cm^−1^ [45]. To explicate the role of the EG additive in the NaCl/H_2_O/EG hybrid electrolytes, Raman spectroscopy was performed on the various electrolyte formulations (Figure 3e,f). The Raman spectra revealed that upon the addition of EG, a COH bending peak emerged at 1464 cm^−1^ (within the range of 1450–1470 cm^−1^) for the EG2 electrolyte, which exhibited a slight blue shift to 1469 cm^−1^ (EG8). Additionally, the CH stretching observed at 2940 cm^−1^ underwent a minor red shift to 2934 cm^−1^, suggesting the formation of hydrogen bonds between the hydrogen atoms of water and the oxygen atoms in EG [46]. Concurrently, the OH stretching of water at 3431 cm^−1^ shifted to a lower wavenumber of 3400 cm^−1^ (EG8), further substantiating the formation of hydrogen bonds between EG and water molecules. The interaction between EG and water molecules partially disrupts the hydrogen-bonding network among water molecules, attenuating their reactivity, which consequently diminishes the occurrence of hydrogen and oxygen evolution side reactions, thereby contributing to the enhanced stability of the electrolyte [47].

### 2.3. Electrochemical Performance of MXene MSCs

**Figure 4:**
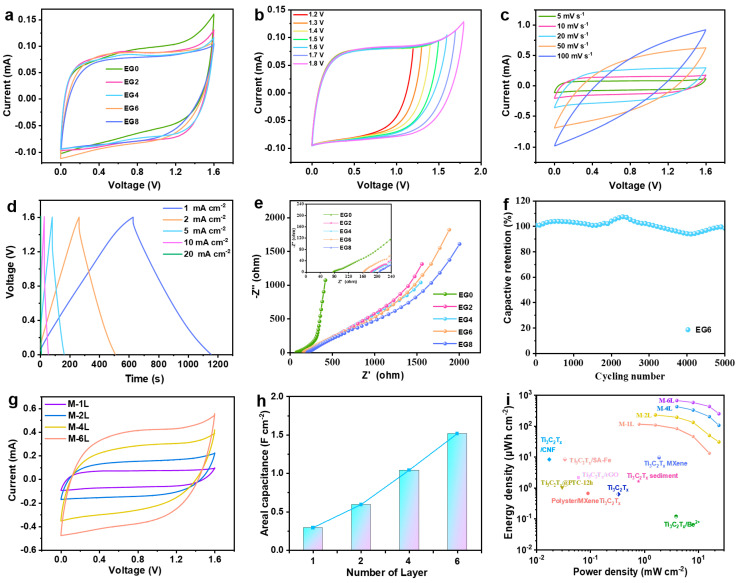
Electrochemical performance of MXene MSCs: (**a**) CV curves of MSC-X; (**b**) CV curves of MSC-6 at varied cut-off voltages; (**c**) CV curves and (**d**) GCD profiles of MSC-6; (**e**) EIS Nyquist plot for MSC-X; (**f**) cycling stability of MSC-6 at 10 mA cm^−2^; (**g**) CV curves and (**h**) corresponding areal capacitance at 5 mV s^−1^ for M-YL; (**i**) Ragone plot depicting comparative energy and power densities of M-YL with previously reported MXene-based MSCs.

In a comprehensive endeavor to elucidate the influence of varying NaCl/H_2_O/EG hybrid electrolytes on the electrochemical performance of MXene MSCs, we subjected these devices—designated as MSC-X where X correlates to the EG composition in the hybrid gel electrolyte (EGX)—to electrochemical characterization at ambient conditions. The CV curves, acquired at a scan rate of 5 mV s^−1^ and depicted in Figure 4a, revealed a pronounced inverse correlation between the EG concentration and the polarization extent. An excessive presence of EG was found to inversely impinge on the capacitance values of the MSCs. This observed interplay was further corroborated by GCD profiles (Figures S4–S7), which highlighted a pronounced limitation in the charge potential of MSC-0 up to 1.2 V, as shown in Figure S4. In contrast, the introduction of EG into the hybrid electrolyte facilitated enhanced charging potentials in MSC-2 and MSC-4, as presented in Figures S5 and S6. However, MSC-8 exhibited a significant decline in both capacitance and rate performance when the proportion of EG surpassed a certain threshold, as evidenced in Figure S7. These findings designate MSC-6 as the optimal configuration, striking an ideal balance between EG content and superior electrochemical performance, suggesting a nuanced interaction between hybrid electrolyte composition and supercapacitor function.

In a comprehensive investigation to delineate the optimal operational parameters of the micro-supercapacitor denoted as MSC-6, CV analyses were meticulously conducted across a range of terminal voltages. Employing a scan rate meticulously set to 5 mV s^−1^ (Figure 4b), the device exhibited remarkable electrochemical stability. The CV curves of MSC-6 retained a quasi-rectangular shape, characteristic of the ideal capacitive behavior, up to an elevated operational ceiling of 1.6 V. It was beyond this threshold that significant electrochemical polarization became apparent (Figure 4c,d), thereby demarcating the maximum operational voltage at an impressive 1.6 V. This is a significant enhancement over traditional voltage limits and highlights the exceptional potential of MSC-6 for energy storage applications. When subjected to galvanostatic charge–discharge at a current density of 1 mA cm^−2^, MSC-6 demonstrated an outstanding areal capacitance of 329 mF cm^−2^ (Figure S8). This performance metric not only significantly exceeds that of the majority of previously documented MXene- and carbon-based MSCs [36,48] but also underscores the high energy storage capacity of MSC-6. Further scrutiny revealed that the integration of EG into the device led to a discernible increase in the internal resistance of the supercapacitors (Figure 4e). This phenomenon is attributed to the consequential decrement in ionic conductivity within the hybrid electrolyte system, which is correlated with the rise in the EG concentration. Such an increase in resistance could potentially impede the ionic transport necessary for charge storage and release. Despite the aforementioned challenge, MSC-6 showcased laudable endurance and robustness in its electrochemical performance. Remarkably, after enduring 5000 charge–discharge cycles at a current density of 10 mA cm^−2^, the device preserved 97% of its initial capacitance, a testament to its durability, as manifested in Figure 4f. This level of cycling stability positions MSC-6 as a compelling candidate for long-term energy storage solutions in advanced electronic devices, where reliability and efficiency are paramount.

To enhance the electrochemical performance of MXene MSCs, a series of devices with incremental active material loadings were meticulously engineered by varying the number of constituent layers (denoted as M-YL, where YL signifies the layer count; Figure S9). These alterations aimed to amplify the areal capacitance and energy density, two pivotal metrics in energy storage efficacy. The CV curves of the M-YL series, obtained at a scan rate of 5 mV s^−1^, were exemplary in their rectangularity, attesting to the expedited charge storage kinetics that these materials facilitate (Figure 4g). The evolution of the areal capacitance in the M-YL series was observed to be nearly proportional to the number of printed layers, culminating in M-6L achieving an exceptional areal capacitance of 1.51 F cm^−2^ (Figure 4h). This progressive increase in capacitance with additional layers signifies a strategic advancement in the fabrication of high-performance MSCs. Nonetheless, a trade-off became evident as the increase in printed layers, which concurrently amplified the electrode thickness, introduced a degree of latency into the charge–discharge dynamics. This increment in thickness was found to slightly attenuate the rate capability of the M-YL MSCs (Figures S10 and S11). Despite this, the electrochemical performance remained robust, as the energy storage capabilities of the devices continued to improve with additional layering. The energy storage prowess of the M-YL series is further elucidated in Figure 4i, where M-1L demonstrates an energy density of 116.8 μWh cm^−2^ at a power density of 0.798 mW cm^−2^. Impressively, when the architecture was scaled up to six layers, M-6L achieved a substantial elevation in energy density, reaching 675 μWh cm^−2^ at a power density of 3.933 mW cm^−2^. This escalation not only underscores the scalability of MXene MSCs but also notably the energy density exceeds those of the majority of previously reported MXene MSCs, as cataloged in Table S1. The results from this investigation vividly underscore the potential for MXene MSCs to be tailored for high-capacity energy storage applications. By judiciously optimizing the layer number, this study achieves a delicate balance between increased energy storage and the maintenance of rapid charge–discharge rates, paving the way for the development of next-generation MSCs that do not compromise power delivery or energy density.

### 2.4. Integrative Electrochemical Performance of MSCs

**Figure 5:**
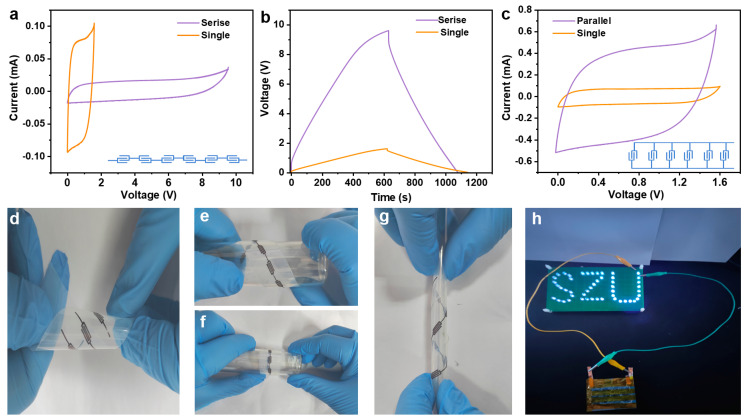
Integrative performance of MSC-6 devices: (**a**) CV curves at 5 mV s^−1^ and (**b**) GCD profiles at 1 mA cm^−2^ of six series-connected MSC-6; (**c**) CV curves of six parallel-connected MSC-6 at 5 mV s^−1^; (**d**–**g**) optical images of MSCs under bending and twisting deformations; (**h**) an “SZU” composed of 49 LEDs powered by an integrated device with three rows of four series-connected MSC-6 in parallel.

In our endeavor to enhance the electrochemical performance of MSCs, we exploited the advanced capabilities of 3D printing technology to fabricate MSC arrays in both series and parallel configurations. Utilizing the exceptional electrical conductivity of MXene-based inks, which act not only as the microelectrode material but also as efficient conductive bridges between individual MSC units, we effectively eliminated the necessity for traditional metal connectors. This strategic design choice appreciably simplifies the overall architecture of the MSC array. The CV curves of six serially connected MSC-6 units unveiled a proportional amplification of the output voltage, achieving a combined potential of 9.6 V, as depicted in Figure 5a. This voltage enhancement was further substantiated by GCD profiles, which demonstrated a consistent charge and discharge duration across the MSCs, thereby confirming the additive nature of the voltage in a series arrangement (Figure 5b). In parallel, the capacitive attributes of six MXene MSCs were significantly bolstered, as evidenced by their GCD profiles (Figure 5c and Figure S12). The amalgamation of MSCs in this manner effectively multiplies the available capacitance, providing a substantial boost in energy storage capacity without compromising the charge/discharge efficiency, thereby illustrating the feasibility of engineering MSCs with superior voltage and capacitance for advanced energy storage applications.

To further elucidate the mechanical flexibility inherent in MXene microelectrodes, we conducted a series of deformation tests on both singular microelectrode units and a sextet configuration wired in series. These assemblies were subjected to dynamic mechanical stresses, including bending and torsional deformation (Figure 5d–g). Throughout this rigorous testing regimen, the deposited conductive paths remained steadfastly anchored to the PET substrate, exhibiting neither signs of detachment nor layer separation—a testament to their exceptional mechanical durability. Moreover, we orchestrated an assembly of MSCs in a hybridized network of series and parallel circuits, which seamlessly supplied power to an “SZU” logo composed of 49 light-emitting diodes (LEDs, Figure 5h). This demonstration not only highlights the modular nature of the MSCs but also their capacity for integration into larger, more complex systems.

## 3. Experimental Section

### 3.1. Fabrication of Ti_3_C_2_T_x_ MXene Ink

MXene (Ti_3_C_2_T*_x_*) was synthesized by selectively etching Ti_3_AlC_2_ utilizing a LiF/HCl etchant solution [49,50]. Initially, 2 g of LiF was thoroughly stirred into 40 mL of 9 M HCl for 30 min. Subsequently, 2 g of Ti_3_AlC_2_ was gradually introduced into the aforementioned solution over approximately 10 min at a controlled temperature of 35 °C, with stirring continued for 24 h. The etched product was then subjected to acid washing with 1 M HCl 2–3 times, followed by repeated deionized water rinsing at a centrifugation speed and duration of 3500 rpm and 5 min, respectively, until the supernatant pH approximated 7. The precipitate was redispersed in 200 mL of deionized water and subjected to ultrasonication for 40 min in an ice bath under an argon atmosphere. The ultrasonicated solution was then centrifuged at 3500 rpm for 1 h to collect the supernatant, yielding an MXene solution. This solution was further centrifuged at 10,000 rpm for 30 min to procure the sediment, which was then centrifuged for 1 h to eliminate excess water, resulting in a printable MXene ink.

### 3.2. Preparation of Gel Electrolyte

Initially, 0.3 g of NaCl was apportioned into five identical borosilicate glass bottles. These vessels were systematically identified using the nomenclature EGX, wherein ‘X’ denotes the series including 0, 2, 4, 6, and 8. Each bottle was then augmented with 5 g of ultrapure deionized water, and a magnetic stirrer was employed to ensure that NaCl was thoroughly solubilized, yielding a uniform 1 m NaCl solution. In a sequential manner, aliquots of EG, specifically 1, 2, 3, and 4 mL in volume, were introduced to the respective bottles, labeled EG2 to EG8. This procedure enabled the precise generation of NaCl/H_2_O/EG hybrid electrolytes of varying compositions, designated as EG2, EG4, EG6, and EG8. The synthesis of the gel electrolyte involved the integration of fumed silicon dioxide (SiO_2_) powder into the mixture at a carefully calculated ratio of 10 wt% by weight relative to the total solvent volume. Continuous stirring was maintained to effectuate a homogeneous dispersion of SiO_2_, ultimately producing NaCl/H_2_O/EG gel electrolytes with consistent viscoelastic properties, suitable for the ensuing electrochemical evaluation and utilization in high-performance energy storage devices.

### 3.3. Fabrication of MSCs via 3D Printing

Symmetrical MSCs were fabricated using a 3D printer (F4200N, FISNAR, Shenzhen, China). The prepared MXene ink was loaded into a 30 mL syringe (specifications: 30 mL, outer diameter: 25.2 mm, inner diameter: 22.5 mm, total length: 118 mm), which was sealed at both ends. The syringe was centrifuged at 3000 rpm for 5 min to eliminate air bubbles. The syringe was then assembled with a needle of 210 µm diameter and connected to a pneumatic fluid dispenser (FISNAR DC50, Shenzhen, China). The 3D printing design, conceived using software (AutoCAD 2023, Autodesk Inc., San Francisco, CA, USA), was transferred to the 3D printer, and the graphic information was translated into printing stage code instructions using vendor-provided software (RoboEdit V2.6, Fisnar, Shenzhen, China). The air pressure and movement speed of the 3D printing device were set at 30 psi and 4 mm s^−1^, respectively, to execute the code program that incrementally deposited the ink onto a commercial polyethylene terephthalate (PET) substrate. The resulting microelectrodes were subjected to a freeze-drying process to preserve their 3D structure. Finally, the requisite gel electrolyte was uniformly applied over the electrodes, and the assembly was encapsulated using polyimide tape to produce the MXene MSCs.

### 3.4. Material Characterization

MXene was analyzed using an X-ray diffractometer (XRD, SmartLab, Rigaku, Tokyo, Japan). The surface morphology and structure of the MXene microelectrodes were investigated with a field-emission scanning electron microscope (FESEM, SU1510, HITACHI, Tokyo, Japan). The morphology of MXene was observed and analyzed by transmission electron microscopy (TEM, TECNAI G2F20, FEI, Amsterdam, The Netherlands). The molecular vibrational information of MXene and the NaCl/H_2_O/EG hybrid electrolytes with varying EG concentrations was characterized using a Raman spectrometer (XploRA Plus, HORIBA, Kyoto, Japan) with a laser excitation wavelength of 532 nm. The rheological behavior of the MXene ink was examined using a rheometer (Discovery HR-20, TA Instruments, New Castle, DE, USA). The shear viscosity of the MXene ink as a function of the shear rate was assessed using a rotational/steady-state mode, with the shear rate ranging from 10^−3^ to 10^4^ s^−1^. The storage and loss modulus variations with scanning stress were measured in the oscillatory/dynamic mode at a constant frequency of 1 Hz, across a stress range of 10^−3^ to 10^4^ Pa. Peak hold tests, simulating the extrusion printing process, were conducted at a constant shear rate with intervals (100 s at 0.1 s^−1^, followed by 100 s at 100 s^−1^, and then reverting to 0.1 s^−1^ for 150 s).

### 3.5. Electrochemical Measurements

Electrochemical characterization was meticulously performed to elucidate the dynamic properties of the NaCl/H_2_O/EG hybrid electrolytes with varying EG concentrations. Employing the CS2350H electrochemical workstation from Wuhan Keystar, we executed a suite of precise measurements, including cyclic voltammetry (CV) and galvanostatic charge–discharge (GCD). Further, electrochemical impedance spectroscopy (EIS) was deployed to probe the intrinsic resistance and ion transport mechanisms within the electrolyte matrix. These EIS measurements were conducted at the open-circuit potential, judiciously applying an oscillating signal with a minimal amplitude of 5 mV across an expansive frequency domain spanning from 0.01 Hz to 100 kHz, ensuring a thorough impedance response analysis. To accurately determine the ionic conductivity of the NaCl/H_2_O/EG hybrid electrolytes with differential EG content, a conductivity meter (DDSJ-308A) was utilized, providing precise quantification of the transport properties of the hybrid electrolytes.

## 4. Conclusions

In summary, our work reported a NaCl/H_2_O/EG hybrid electrolyte, providing new insights for the design of environmentally friendly, high-voltage, and low-cost aqueous hybrid electrolytes and marking a leap forward in the development of micro-supercapacitors (MSCs) with both high voltage and cost efficiency. The constructed MSC-6 devices, employing our innovative EG6 gel hybrid electrolyte, achieved a notable operational voltage of 1.6 V. Additionally, these devices showcased remarkable areal capacitance reaching 329 mF cm⁻^2^ and an energy density of 116.8 μWh cm⁻^2^. Leveraging advanced 3D printing technology enabled us to fabricate six-layer thick electrodes, propelling the energy density to a remarkable 675 μWh cm⁻^2^ for our M-6L MSCs. Furthermore, the MSCs exhibited excellent mechanical flexibility, superior module integration, and practicality. The breakthroughs presented here not only extend the voltage threshold of aqueous electrolytes but also open new pathways for scalable, flexible, high-performance energy storage solutions. The impressive mechanical flexibility and integration potential of these MSCs underscore their suitability for hybrid-integrated systems on a chip for flexible electronics, next-generation self-powered wearable electronics, and IoT devices.

## Data Availability

Data are contained within the article and Supplementary Materials.

## Supplementary Materials

The following supporting information can be downloaded at: https://www.mdpi.com/article/10.3390/molecules29061393/s1, Figure S1: Optical images of aqueous MXene ink: (a) normal and (b)inverted orientations. Figure S2: Print the specific parameters of the electrode. Figure S3: Optical images of EG/NaCl electrolytes with varying amounts of EG. Figure S4: Electrochemical performance of MSCs-0. (a) CV curves at different scan rates and (b) GCD profiles at various current densities. Figure S5. Electrochemical performance of MSCs-2. (a) CV curves at different scan rates and (b) GCD profiles at various current densities. Figure S6. Electrochemical performance of MSCs-4. (a) CV curves at different scan rates and (b) GCD profiles at various current densities. Figure S7. Electrochemical performance of MSCs-8. (a) CV curves at different scan rates and (b) GCD profiles at various current densities. Figure S8. The actual capacitance of MSCs-6 in different test methods: (a) scan rate and (b) current density. Figure S9. Schematic representation of MXene-MSCs with different printed layers. Figure S10. Electrochemical properties of MXene-MSCs with different printed layers. CV curves of (a) M-2L, (b) M-4L, and (c) M-6L at different scan rates. GCD profiles of (d) M-2L, (e) M-4L, and (f) M-6L at various current densities. Figure S11. Specific capacitance of MXene-MSCs with different printed layers at scan rates ranging from 5 to 100 mV s^−1^. Figure S12. GCD profiles of six parallel MSCs-6 at a current density of 2 mA cm^−2^. Table S1. Comparison of the energy density and power density of MXene-based supercapacitors between this work and previous work. References [48,51,52,53,54,55,56,57,58] have been cited in the Supplementary Materials.
