# Supplementary material for: Broadening the Voltage Window of 3D-Printed MXene Micro-Supercapacitors with a Hybridized Electrolyte
Source: Molecules. 2024 Mar 20;29(6):1393. doi: 10.3390/molecules29061393 (PMC10974977; doi:10.3390/molecules29061393)
Supplement: Supplementary file 1 [file molecules-29-01393-s001.zip › molecules-2902927-supplementary.pdf]

Supporting Information

Broadening the Voltage Window of 3D-Printed MXene Micro-Supercapacitors with a Hybridized Electrolyte

Xin Jiang [1,2], Haowen Jia [1], Xuan Chen [1], Jiajia Li [1], Yanling Chen [1], Jin Jia [1,*], Guangzhen Zhao [1], Lianghao Yu [1], Guang Zhu [1,*] and Yuanyuan Zhu [1,*]

[1] Key Laboratory of Spin Electron and Nanomaterials of Anhui Higher Education Institutes, Suzhou University, Suzhou 234000, China; jiangxinty@163.com (X.J.); jiahaowen100823@163.com (H.J.); cxuan0221@163.com (X.C.); lijiajia040326@163.com (J.L.); chenyanl0516@163.com (Y.C.); zhaogzgold@126.com (G.Z.); yulianghao789@163.com (L.Y.)

[2] School of Mechanics and Optoelectronic Physics, Anhui University of Science and Technology, Huainan 232001, China

* Correspondence: jiajin@ahszu.edu.cn (J.J.); guangzhu@ahszu.edu.cn (G.Z.); zhuyy@ahszu.edu.cn (Y.Z.)

Calculations:

The capacitance of the cross-linked electrode can be calculated using the cyclic voltammetry (CV) and galvanostatic charge-discharge (GCD) curves. The specific calculation formulas are provided below:

$$C_{CV} = \frac{1}{v \times \Delta V} \int I(V)dV \qquad (S1)$$

$$C_{GCD} = \frac{I \times \Delta t}{\Delta V} \qquad (S2)$$

The current during the cyclic voltammetry (CV) tests, denoted as I(V) (mA), was measured. The scan rate, represented as v (V s^{-1}), and the discharge current, denoted as I (mA), were recorded. The discharge time, denoted as Δt (s), and the voltage range of the discharge, denoted as ΔV (V), were also noted.

The calculation formula for the specific capacitance of the dual-electrode device is provided below:

$$C_{areal} = \frac{C}{A_{device}} \qquad (S3)$$

The areal capacitance of the dual-electrode device, denoted as C_{areal} (mF cm^{-2}), was determined, while A_{device} (cm^{-2}) represents the electrode area of the dual-electrode configuration.

The calculation formulas for the areal energy density, E_A (μWh cm^{-2}) (Equation (S4)), and the areal power density, P_A (μW cm^{-2}) (Equation (S5)), of the device are provided as follows:

$$E_A = \frac{C_{areal}}{2 \times 3600} \times \Delta V^2 \qquad (S4)$$

$$P_A = \frac{E_A}{\Delta t} \times 3600 \qquad (S5)$$

Here, ΔV (V), Δt (s), and C_{areal} (mF cm^{-2}) are as described above.

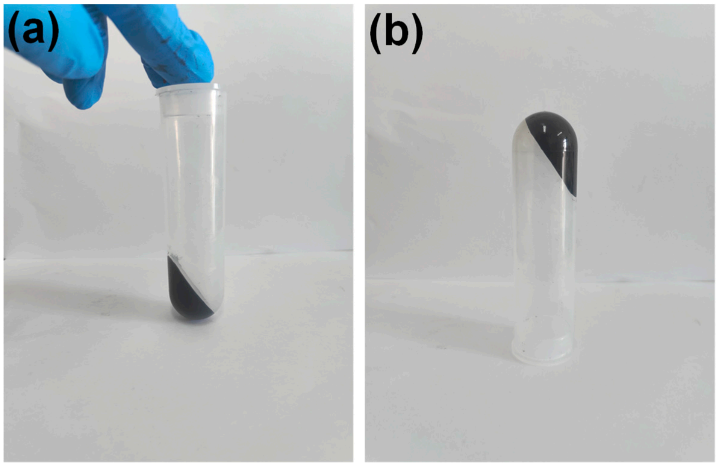

Figure S1. Optical images of aqueous MXene ink: (a) normal and (b)inverted orientations.

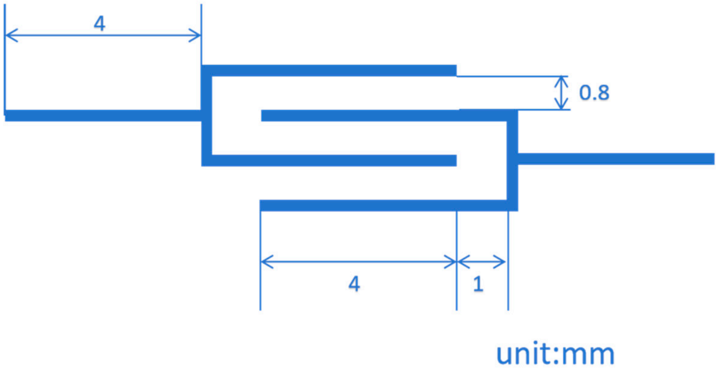

Figure S2. Print the specific parameters of the electrode.

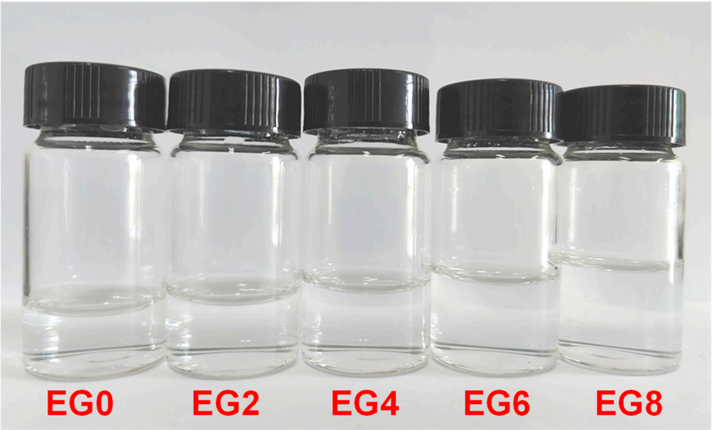

Figure S3. Optical images of EG/NaCl electrolytes with varying amounts of EG.

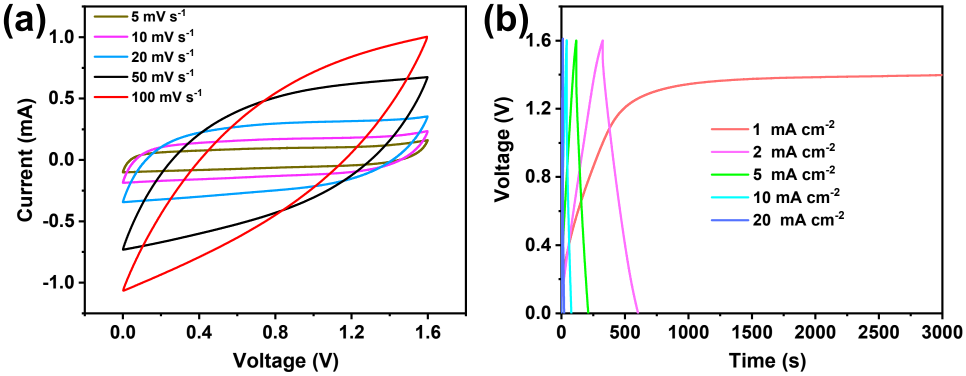

Figure S4. Electrochemical performance of MSCs-0. (a) CV curves at different scan rates and (b) GCD profiles at various current densities.

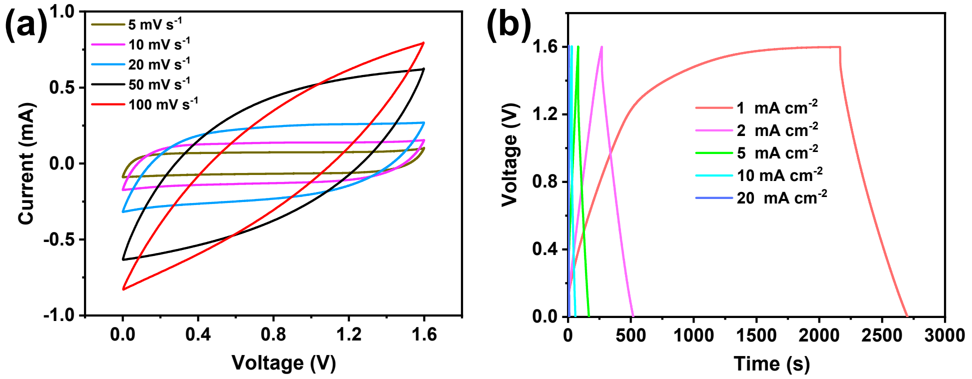

Figure S5. Electrochemical performance of MSCs-2. (a) CV curves at different scan rates and (b) GCD profiles at various current densities.

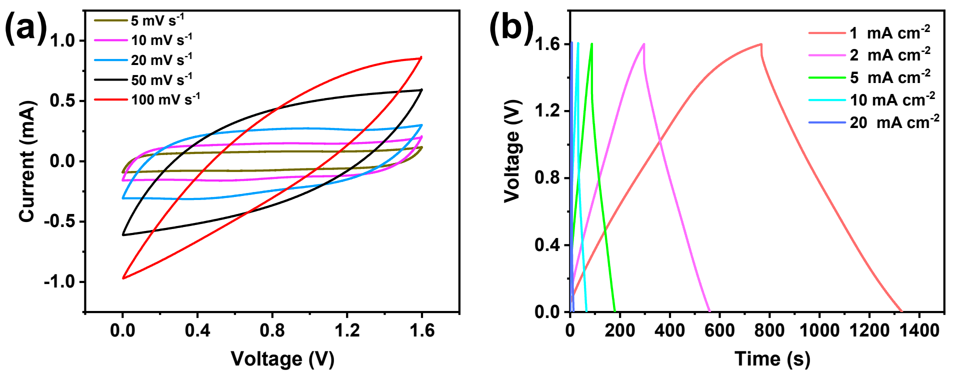

Figure S6. Electrochemical performance of MSCs-4. (a) CV curves at different scan rates and (b) GCD profiles at various current densities.

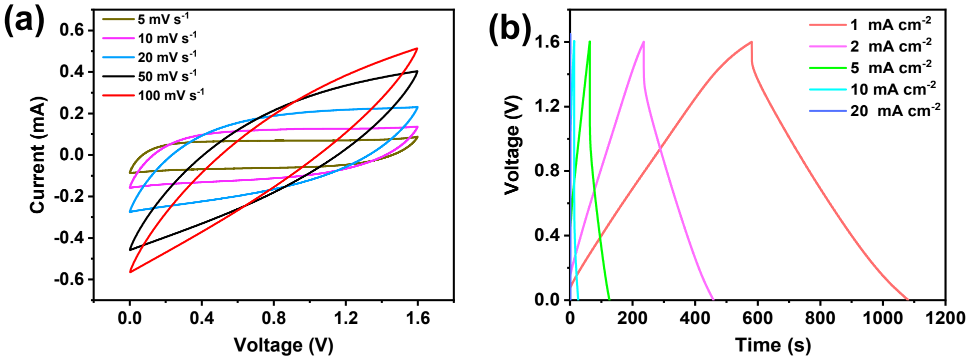

Figure S7. Electrochemical performance of MSCs-8. (a) CV curves at different scan rates and (b) GCD profiles at various current densities.

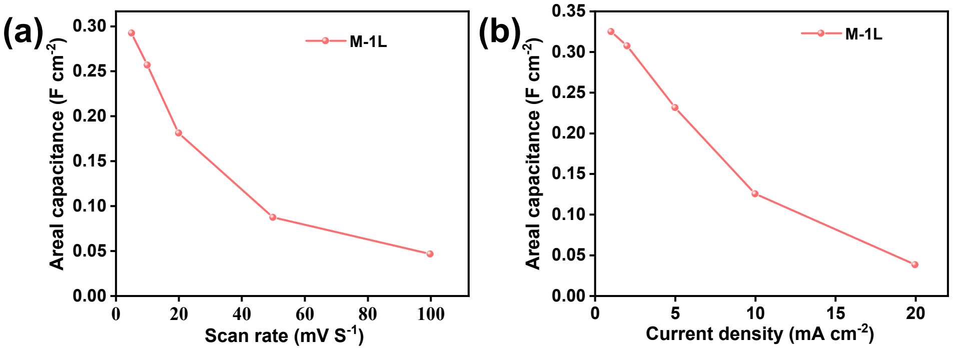

Figure S8. The actual capacitance of MSCs-6 in different test methods: (a) scan rate and (b) current density.

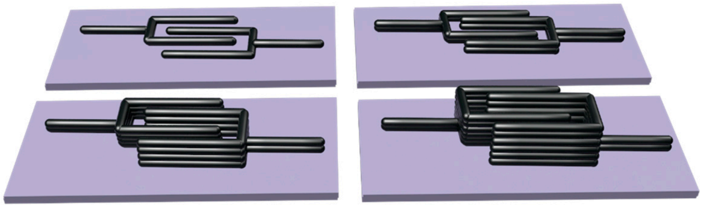

Figure S9. Schematic representation of MXene-MSCs with different printed layers.

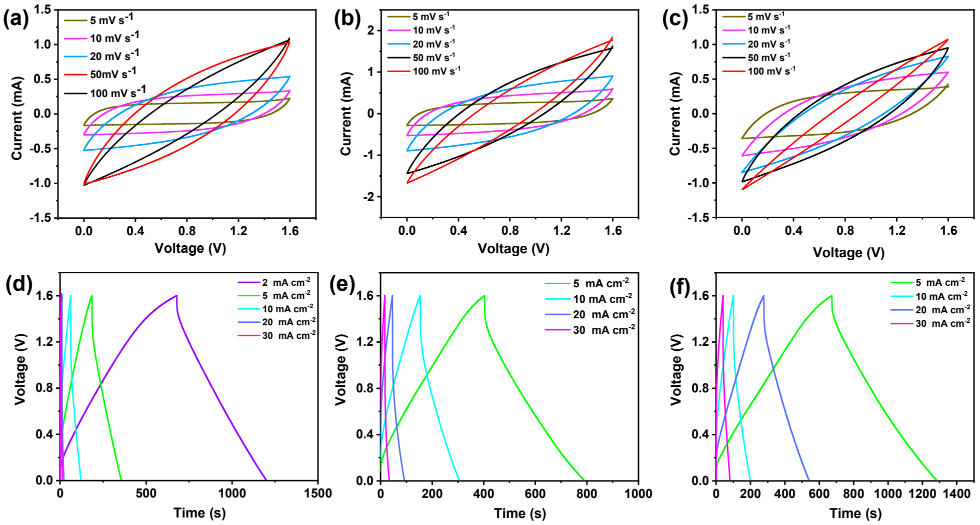

Figure S10. Electrochemical properties of MXene-MSCs with different printed layers. CV curves of (a) M-2L, (b) M-4L, and (c) M-6L at different scan rates. GCD profiles of (d) M-2L, (e) M-4L, and (f) M-6L at various current densities.

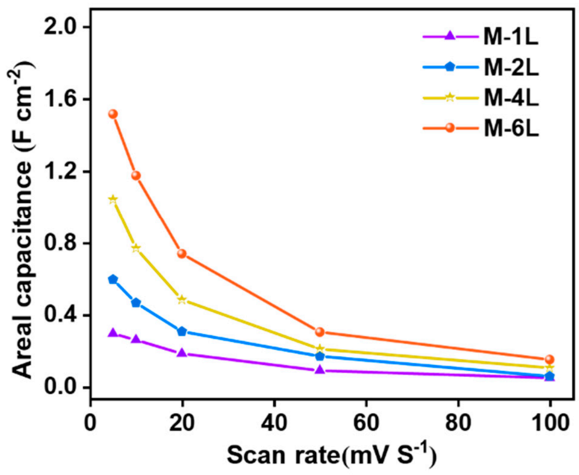

Figure S11. Specific capacitance of MXene-MSCs with different printed layers at scan rates ranging from 5 to 100 mV s^{-1}.

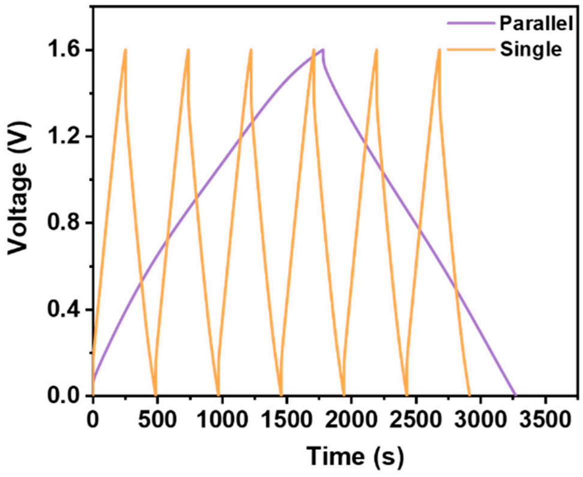

Figure S12. GCD profiles of six parallel MSCs-6 at a current density of 2 mA cm^{-2}.

Table S1. Comparison of the energy density and power density of MXene-based supercapacitors between this work and previous work

Materials	Electrolytes	Voltage (V)	C_A (mF cm^{-2})	Energy density (μWh cm^{-2})	Power density (mW cm^{-2})	Refs.
$Ti_3C_2T_x$/rGO	PVA/H_2SO_4	0.6	34.6	2.18	0.06	[1]
$Ti_3C_2T_x$/Be^{2+}	$ZnSO_4$-gel	0.6	77.2	0.12	3.86	[2]
$Ti_3C_2T_x$/SA-Fe	PVA/H_2SO_4	0.6	123.8	8.44	0.034	[3]
$Ti_3C_2T_x$ sediment	PVA/H_2SO_4	0.6	158	1.64	0.778	[4]
$Ti_3C_2T_x$ MXene	PVA/H_3PO_4	0.6	~225	9.7	1.875	[5]
Polyster/MXene $Ti_3C_2T_x$	PVA/H_2SO_4	0.6	18.39	0.67	0.09	[6]
$Ti_3C_2T_x$/CNT	PVA/H_2SO_4	0.6	30.76	8.37	0.017	[7]
$Ti_3C_2T_x$	PVA/H_2SO_4	0.6	61	0.63	0.33	[8]
$Ti_3C_2T_x$@PTC-12h	PVA/H_2SO_4	0.6	20.8	1.04	0.03	[9]
M-1L	1 m NaCl/EG	1.6	329	116.8	0.798	This work
M-6L	1 m NaCl/EG	1.6	1903	675	3.933	This work

PTC：polylactic acid (PLA) and hermoplastic polyurethane (TPU) as a matrix and carbon black (CB).

References:

1. Yue, Y.; Liu, N.; Ma, Y.; Wang, S.; Liu, W.; Luo, C.; Zhang, H.; Cheng, F.; Rao, J.; Hu, X.; Su, J.; Gao, Y., Highly self-healable 3D microsupercapacitor with MXene-graphene composite aerogel. *ACS Nano* **2018**, 12, (5), 4224-4232.
2. Li, S.; Shi, Q.; Li, Y.; Yang, J.; Chang, T. H.; Jiang, J.; Chen, P. Y., Intercalation of metal ions into $Ti_3C_2T_x$ MXene electrodes for high-areal-capacitance microsupercapacitors with neutral multivalent electrolytes. *Adv. Funct. Mater.* **2020**, 30, (40), 2003721.
3. Wang, G.; Zhang, R.; Zhang, H.; Cheng, K., Aqueous MXene inks for inkjet-printing microsupercapacitors with ultrahigh energy densities. *J. Colloid Interface Sci.* **2023**, 645, 359-370.
4. Abdolhosseinzadeh, S.; Schneider, R.; Verma, A.; Heier, J.; Nuesch, F.; Zhang, C. J., Turning trash into treasure: additive free MXene sediment inks for screen-printed micro-supercapacitors. *Adv. Mater.* **2020**, 32, (17), 2000716.
5. Shao, Y.; Wei, L.; Wu, X.; Jiang, C.; Yao, Y.; Peng, B.; Chen, H.; Huangfu, J.; Ying, Y.; Zhang, C. J.; Ping, J., Room-temperature high-precision printing of flexible wireless electronics based on MXene inks. *Nat. Commun.* **2022**, 13, (1), 3223.
6. Shao, W.; Tebyetekerwa, M.; Marriam, I.; Li, W.; Wu, Y.; Peng, S.; Ramakrishna, S.; Yang, S.; Zhu, M., Polyester@MXene nanofibers-based yarn electrodes. *J. Power Sources* **2018**, 396, 683-690.
7. Zhao, J.; Zhang, Y.; Huang, Y.; Zhao, X.; Shi, Y.; Qu, J.; Yang, C.; Xie, J.; Wang,

J.; Li, L.; Yan, Q.; Hou, S.; Lu, C.; Xu, X.; Yao, Y., Duplex printing of all-in-one integrated electronic devices for temperature monitoring. *J. Mater. Chem. A* **2019**, 7, (3), 972-978.

8. Zhang, C.; Kremer, M. P.; Seral-Ascaso, A.; Park, S. H.; McEvoy, N.; Anasori, B.; Gogotsi, Y.; Nicolosi, V., Stamping of flexible, coplanar micro-supercapacitors using MXene inks. *Adv. Funct. Mater.* **2018**, 28, (9), 1705506.

9. Zhu, G.; Hou, Y.; Lu, J.; Zhang, H.; Zhuang, Z.; Baig, M. M.; Khan, M. Z.; Akram, M. A.; Dong, S.; Liu, P.; Ge, X.; Zhang, Y., MXene decorated 3D-printed carbon black-based electrodes for solid-state micro-supercapacitors. *J. Mater. Chem. A* **2023**, 11, (46), 25422-25428.
